# Transcranial Magnetic Stimulation for the treatment of tinnitus: Effects on cortical excitability

**DOI:** 10.1186/1471-2202-8-45

**Published:** 2007-07-02

**Authors:** Berthold Langguth, Tobias Kleinjung, Joerg Marienhagen, Harald Binder, Philipp G Sand, Göran Hajak, Peter Eichhammer

**Affiliations:** 1 Department of Psychiatry, Psychosomatics and Psychotherapy, University of Regensburg, Universitaetsstraße 84, 93053 Regensburg, Germany; 2 Department of Otorhinolaryngology and Audiology, University of Regensburg, Franz-Josef-Strauß-Allee 11, 93053 Regensburg, Germany; 3 Department of Nuclear Medicine, University of Regensburg, Franz-Josef-Strauß-Allee 11, 93053 Regensburg, Germany; 4 Department of Medical Biometry and Statistics, University of Freiburg, Stefan-Meier-Strasse 26, 79104 Freiburg, Germany

## Abstract

**Background:**

Low frequency repetitive transcranial magnetic stimulation (rTMS) has been proposed as an innovative treatment for chronic tinnitus. The aim of the present study was to elucidate the underlying mechanism and to evaluate the relationship between clinical outcome and changes in cortical excitability. We investigated ten patients with chronic tinnitus who participated in a sham-controlled crossover treatment trial. Magnetic-resonance-imaging and positron-emission-tomography guided 1 Hz rTMS were performed over the auditory cortex on 5 consecutive days. Active and sham treatments were separated by one week. Parameters of cortical excitability (motor thresholds, intracortical inhibition, intracortical facilitation, cortical silent period) were measured serially before and after rTMS treatment by using single- and paired-pulse transcranial magnetic stimulation. Clinical improvement was assessed with a standardized tinnitus-questionnaire.

**Results:**

We noted a significant interaction between treatment response and changes in motor cortex excitability during active rTMS. Specifically, clinical improvement was associated with an increase in intracortical inhibition, intracortical facilitation and a prolongation of the cortical silent period. These results indicate that intraindividual changes in cortical excitability may serve as a correlate of response to rTMS treatment.

**Conclusion:**

The observed alterations of cortical excitability suggest that low frequency rTMS may evoke long-term-depression like effects resulting in an improvement of subcortical inhibitory function.

## Background

Subjective tinnitus is characterized by the perception of sound or noise in the absence of any internal or external acoustical stimulation. For 1–2% of the general population, this condition causes a considerable amount of distress and interferes seriously with the individual's ability to lead a normal life [1].

The advent of modern neurophysiological and imaging tools has greatly benefited our understanding of the abnormal functioning of the central nervous system as a major cause of chronic tinnitus [2,3]. This is illustrated by a) an enhanced activation of the central auditory system in subjects suffering from tinnitus [4-7], b) the modulation of tinnitus perception by electrical stimulation of the auditory cortex [8,9] and c) changes in the tonotopic map of the auditory cortex visualized with magnetic source imaging [10,11]. These results have been complemented by evidence of dysfunctional thalamocortical processing in tinnitus [12-15]. Additional support for these findings comes from MRI studies demonstrating changes of thalamic structural plasticity in affected subjects [16]. Tinnitus-related hyperexcitability in specific brain regions along with dysfunctional neuroplasticity in critical cortical circuits have paved the way for addressing auditory phantom perceptions with rTMS based protocols: This method uses an electromagnet placed on the scalp that generates magnetic field pulses of very short duration (100–300 μs) and approximately 1.5–2.0 T in strength. After passing largely undistorted through the scalp and scull, the magnetic field induces an electrical current in superficial cortical neurons, which in turn results in neuronal depolarisation [17]. When used in the low-frequency range, rTMS modulates brain activity both in directly stimulated regions and in functionally connected brain areas [18,19]. Thereby it may modulate neuroplasticity in cortical circuits and thalamocortical networks alike [20,21]. rTMS has also been shown to effectively reduce auditory hallucinations in patients with schizophrenia [22,23]. Extending these studies to tinnitus, we have demonstrated that PET- and MRI-guided neuronavigated low-frequency rTMS over the hyperactive auditory cortex can alleviate symptom severity in this phantom sensation [24] as well. A subsequent controlled trial of 14 patients with chronic tinnitus confirmed a significant reduction in tinnitus severity scores after five days of active rTMS as compared to sham treatment [25]. In the majority of patients these beneficial effects remained stable up to six months post rTMS intervention, suggesting the possible induction of long-lasting neuroplastic changes [21,26].

The objective of the present study was to evaluate whether subjective effects of altered tinnitus sensation after rTMS treatment are accompanied by changes in objective data assessing cortical excitability. For this purpose we used transcranial magnetic stimulation (TMS) to serially assess multiple parameters of motor cortical excitability in patients who participated in a sham controlled crossover trial of rTMS [25]. Our testing included (a) motor threshold (MT), which reflects membrane related neuronal excitability; (b) the cortical silent period (CSP), i.e. a correlate of inhibitory function within cortical and subcortical structures; (c) intracortical inhibition (ICI) and (d) intracortical facilitation (ICF), i.e. two tests of intracortical inhibitory and excitatory mechanisms [27].

As has been shown by our group and by other investigators, changes in cortical excitability may serve as a correlate of response to treatment [26,28-32]. Related parameters are sensitive to practice-dependent and deafferentation-induced plastic change in human cortex [33,34] and hold promise for elucidating the underlying mechanisms [35]. Dense functional connections between the central auditory system and the sensorimotoric system are well-known [36,37], and make tinnitus amenable to triggering or modulating by input from sensorimotoric systems [38-42]. To judge by its occurrence in the majority of tinnitus patients [43,44], somatosensoric modulation seems to represent a fundamental attribute of tinnitus [3]. These functional connections may provide the physiological basis for the detection of changes in auditory processing by measuring motor cortex excitability [26,45-47].

## Results

**Figure 1:**
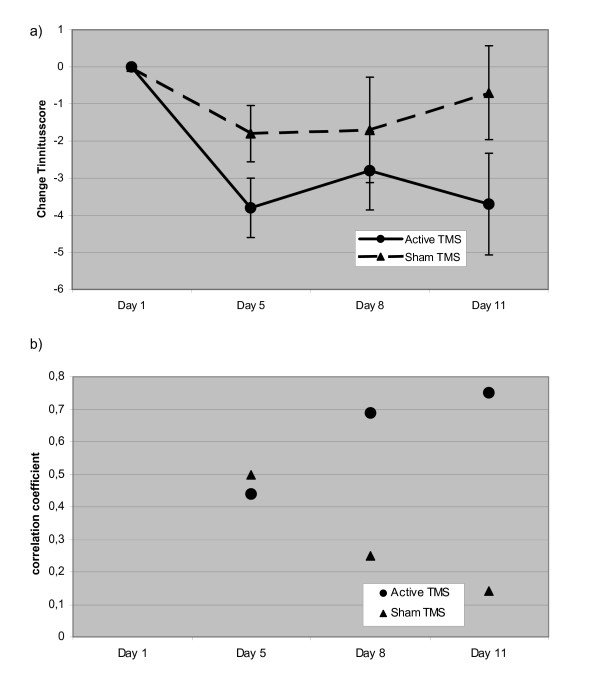
**Changes in tinnitus score and in multiple correlation coefficient during active and sham rTMS**. a) mean reduction in tinnitus scores(ΔTQ) after active and sham rTMS is demonstrated. Error bars represent standard errors. b) multiple correlation coefficient between changes of excitability (ΔE) and changes in tinnitus score (ΔTQ) after active and sham stimulation. Clinical effect (ΔTQ) and correlation of ΔTQ and ΔE were strongest on day 11 (i.e. 6 days post intervention).

**Figure 2:**
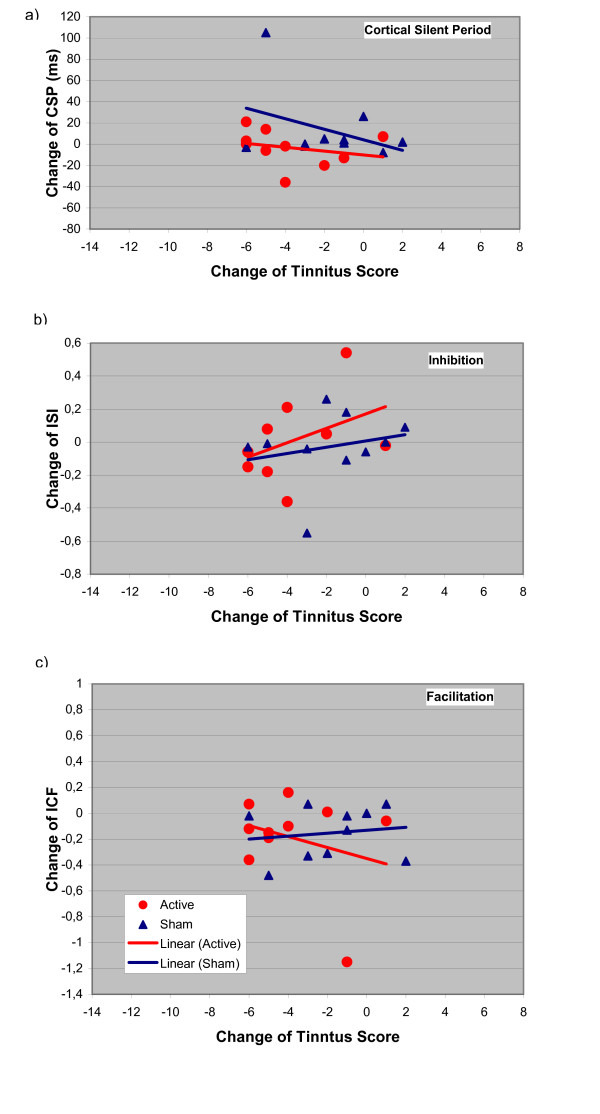
**Alteration of cortical excitability parameters at day 5**. x axis: Tinnitus scores on day 5 compared with day 1 (Negative values correspond to a reduction in tinnitus severity); y axis: a) Changes of the cortical silent period (CSP), b) intracortical inhibition (ISI 2–5 ms) and c) intracortical facilitation (ISI 7–20 ms) relative to baseline. Negative scores for inhibition correspond to an increase in intracortical inhibition.

**Figure 3:**
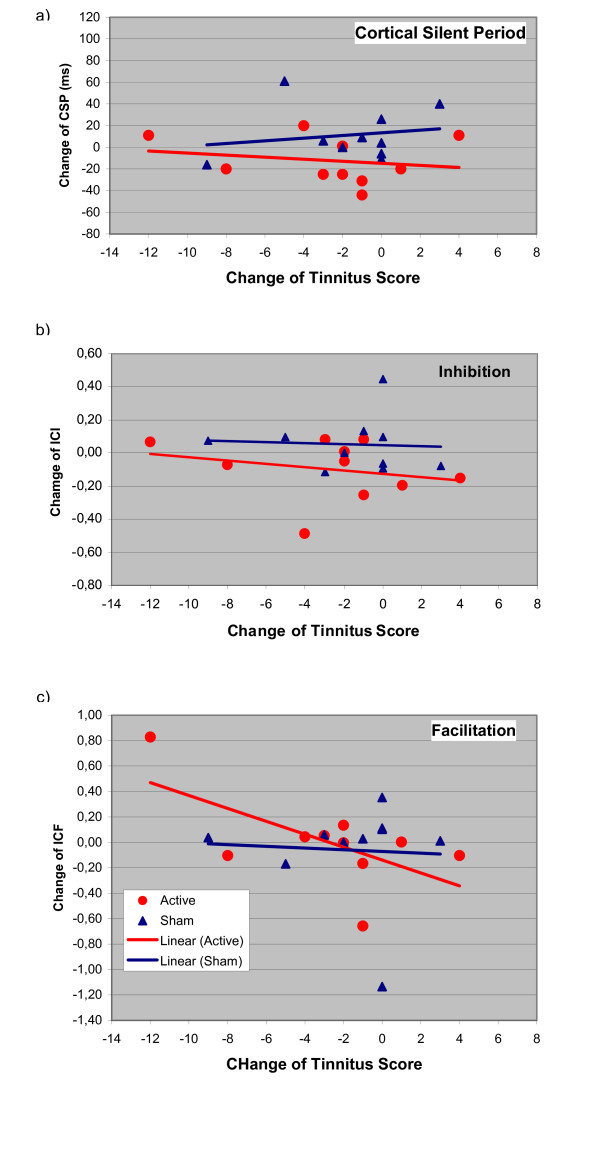
**Alteration of cortical excitability parameters at day 8**. x axis: Tinnitus scores on day 8 compared with day 1 (Negative values correspond to a reduction in tinnitus severity); y axis: a) Changes of the cortical silent period (CSP), b) intracortical inhibition (ISI 2–5 ms) and c) intracortical facilitation (ISI 7–20 ms) relative to baseline. Negative scores for inhibition correspond to an increase in intracortical inhibition.

**Figure 4:**
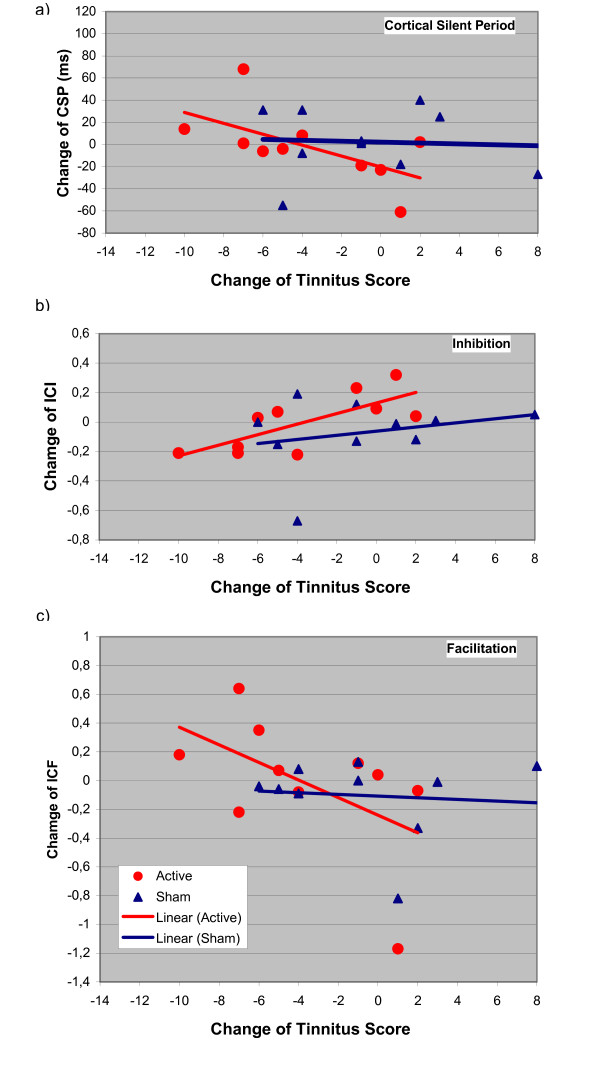
**Alteration of cortical excitability parameters at day 11**. x axis: Tinnitus scores on day 11 compared with day 1 (Negative values correspond to a reduction in tinnitus severity); y axis: a) Changes of the cortical silent period (CSP), b) intracortical inhibition (ISI 2–5 ms) and c) intracortical facilitation (ISI 7–20 ms) relative to baseline. Negative scores for inhibition correspond to an increase in intracortical inhibition. Reduction of the tinnitus score after active rTMS treatment is accompanied by a prolongation of the silent period, an increase in intracortical inhibition and an increase in intracortical facilitation.

All patients completed the study and adverse effects were not observed. At baseline, no statistically significant differences between the verum and sham condition could be found. Considering the low power for detecting such effects with the given small number of observations, this does not necessarily mean that there are no carry-over effects, but at least in the present context they cannot be detected. The treatment response was variable and ranged from no effect to a marked reduction in tinnitus complaints (table 1). After sham treatment, only a slight and transient reduction of tinnitus was observed (fig 1). Separate analysis of variance models for the absolute levels of tinnitus and for the intracortical excitability data over time show no statistically significant effect for any of the measurements. Therefore, further statistical analysis is focused on the change of correlation over time. Following active stimulation, the multiple correlation of excitability change and reduction of tinnitus strengthened from day 5 to day 11 (fig 1). The changes of different parameters of cortical excitability over days are displayed in figures 2, 3 and 4. On day 11 correlation coefficients of verum and sham treatment differed significantly (p = 0.046). Exploratory analysis of the influence of different excitability parameters revealed that the reduction in TQ was correlated with an increase in ICI (r = 0.74, p = 0.015; by definition, lower values of ICI correspond to enhanced ICI) with a trend towards increased ICF (r = -0.58, p = 0.080) and increased CSP (r = -0.61, p = 0.063) (fig 4). In contrast, after sham stimulation, no significant correlation was noted between tinnitus change and altered excitability parameters (ICI r = 0.26, p = 462; ICF r = -0.114, p = 0.754; CSP r = 0.03, p = 0.932) (see fig 4).

**Table 1:** Demographic and clinical data

Gender	Age (yr)^1^	Handedness	Tinnitus Latera-lity^2^	Side of PET activation^3^	Duration (months)^4^	Order of stimula-tion^5^	Tinnitus score before active rTMS^6^	Tinnitus score before sham rTMS^6^	ΔTQ Active rTMS^7^	ΔTQ Sham rTMS^7^
m	61	R	L > R	L	90	A, S	45	37	-7	2
m	48	R	L > R	L > R	12	A, S	42	40	-4	-5
w	48	R	L	R	36	S, A	65	57	-6	+8
m	61	R	L = R	L	140	A, S	74	67	-10	-1
m	59	R	L	L	30	A, S	30	16	-7	-4
m	49	R	L = R	L > R	28	S, A	41	49	-1	-4
m	20	R	L = R	L	6	S, A	40	34	-5	+2
m	29	R	R	L > R	17	S, A	28	25	0	+3
w	41	L	L	L > R	60	A, S	60	61	+2	-1
m	60	L	R	R	48	S, A	47	52	+1	-6

^1^age in years at study begin;

^2 ^tinnitus laterality as reported by the patient; L: left R: right;

^3 ^side of hypermetabolic activity in the FDG PET (the side of increased activity was target for rTMS stimulation);

^4 ^duration of tinnitus in months at study begin;

^5 ^the order of stimulation according to randomization: "A, S" stands for active rTMS first and sham rTMS second and "S, A" for the inverse order;

^6 ^Tinnitus scores as revealed by the Tinnitus Questionnaire of Goebel and Hiller (TQ) [73];

^7 ^ΔTQ: treatment response, indicated as the difference between tinnitus scores on day 11 and day 1 for active rTMS and sham rTMS, respectively.

## Discussion

The above findings from serial measurements of motor cortex excitability in ten patients suffering from chronic tinnitus confirm the interplay of physiological parameters and a subjective reduction in tinnitus complaints after rTMS treatment. This interplay was specific to the verum intervention and has several implications for the etiology and treatment of the condition. However, the results have to be considered with caution, because the small number of subjects increases the danger of overfitting when calculating the multiple correlation coefficients, upon which interpretation is based.

The observed alterations of cortical excitability could be either directly induced by TMS treatment or mediated indirectly, e.g. by changes of patients' motor behaviour after TMS-induced tinnitus improvement. However, changes in tinnitus complaints occurred both in the active and the sham condition, whereas the correlation between tinnitus improvement and alterations of excitability was present only in the active condition. This favours the notion of a direct relationship between rTMS treatment and alterations of cortical excitability. This interpretation is in line with recent studies suggesting that intraindividual changes in cortical excitability may serve as a correlate of response to treatment [26,28-32]. Thus our results give further support to functional connections between the central auditory system and the motor cortex in tinnitus patients as previously described both at the structural level [36] and at the functional level in affected subjects [38,42,45]. Reactivation of the extralemniscal auditory pathway along with the consecutive activation of non-auditory brain areas in some forms of tinnitus [3] may make tinnitus pathophysiology amenable to investigation by measuring motor cortex excitability.

The observed changes in the excitability pattern might reflect neurobiological effects of rTMS that are associated with tinnitus improvement and thus may help to identify the underlying mechanism. Both TMS-induced tinnitus reduction as well as the correlation with cortical excitability were most pronounced six days post active rTMS (fig 1). This delay in clinical and cortical response could reflect the temporal dynamics of rTMS-induced neuroplastic changes, as has been suggested previously [26]. Sustained stimulation effects have already been shown in animal experiments, in which electrical stimulation with 1 Hz administered daily for two weeks induced long-term-depression that outlasted the treatment by at least two weeks [48]. With regard to these delayed stimulation effects, the interval of 9 days between stimulation conditions may have been too short to rule out potential carry-over effects. To safeguard against this potentially confounding factor, we included treatment order in the statistical analysis and tested for baseline differences between stimulation conditions. However, it has to be considered that, due to the small number of patients, the ability to detect such effects is limited.

With respect to different parameters of cortical excitability, tinnitus improvement after low frequency rTMS was related to an extension of the cortical silent period and an increase in both intracortical inhibition and intracortical facilitation. Similar neurophysiological effects have been described after application of GABA_B _agonists [49,50]. In accordance with our findings, prior TMS studies have also demonstrated an extension of the cortical silent period after low frequency rTMS in healthy controls [51], in patients with writer's cramp [52] and schizophrenic patients with auditory hallucinations [29]. Prolongation of the cortical silent period is believed to reflect improved inhibitory function within cortical and subcortical structures, including the thalamus [27,53]. In light of this research, the clinical improvement induced by rTMS may be related to enhanced GABA_B _inhibitory function at the subcortical level. Strong support for the notion that low frequency rTMS modulates thalamocortical networks comes from a recent neuroimaging study, which has demonstrated neuroplastic changes in the temporal cortex and in the thalamus after 1 Hz rTMS [21]. Finally, animal studies testify to the inhibition of relay cells mediated by GABAergic neurons in the reticular nucleus (RTN) of the thalamus following electrical stimulation of corticothalamic fibers [54,55]. This inhibition can reach wide parts of the thalamus, including auditory thalamic neurons [56,57]. Thus, similar to electrical stimulation, low frequency rTMS may reduce cortical excitability by activating inhibitory GABAergic neurons in the thalamic reticular nucleus.

TMS-induced modulation of corticothalamic pathways may also explain the alteration of intracortical excitability (ICI and ICF) observed: Stimulation of corticothalamic pathways in animals has been shown to induce long-term depression (LTD) within the auditory cortex via activation of type1-metabotropic glutamatergic receptors [58]. Such induction of LTD entrains moderate activation of N-methyl-D-aspartate (NMDA)-mediated excitatory circuits [58]. Enhancement of N-methyl-D-aspartate (NMDA)-transmission, in turn, is reflected by increased ICF according to pharmacological studies [33,59].

In this context, the association observed between increased ICF and reduced tinnitus after low frequency rTMS may reflect NMDA-mediated LTD induction. This hypothesis is supported by TMS studies in humans, which demonstrated the induction of neuroplasticity by low frequency rTMS. When areas of locally increased excitability were stimulated, 1 Hz rTMS had a pronounced down-regulating effect, which outlasted the stimulation period, suggesting long-term depotentiation as the most relevant biological factor behind rTMS effects [33,60].

In the present study, the use of a neuronavigation system ensured that rTMS was performed exactly over hypermetabolic brain areas. As metabolic hyperactivity of the primary auditory cortex in tinnitus patients is presumed to reflect enhanced synaptic transmission associated with disinhibition, low frequency rTMS may have selectively depotentiated enhanced synaptic weights [61].

## Conclusion

Low frequency rTMS over the hyperactive auditory cortex has repeatedly been shown to reduce tinnitus sensations [24-26,62,63], however treatment results have been difficult to predict in individual subjects. In the study presented here, we delineate an association between clinical improvement and alteration of cortical excitability. The changes observed in different parameters of cortical excitability are consistent with the hypothesis that clinical effects of low frequency rTMS are dependent on corticothalamic processing [21].

Our findings may help to explain the variability in clinical outcome on the basis of an individual response in cortical physiology [64,65]. With regard to future interventions, measurements of cortical excitability with ppTMS hold promise as a neurophysiological marker of rTMS induced neuroplasticity. This should allow tailored treatment strategies to develop that take differences in genetic background and behavioural state into account, both of which affect the induction of neuroplastic changes [66-68].

We are aware of the limitations of our data, as they result from a pilot study with a relatively low number of subjects. Further investigations with longer observation periods and larger collectives including healthy controls will be necessary to replicate our findings.

## Methods

### Patients

We studied ten patients (8 men, 2 women, mean age 47.7 years; SD 14.2) suffering from mild to severe unilateral or bilateral chronic tinnitus, who participated in a sham controlled rTMS treatment trial. Imaging and clinical results for all patients have already been reported elsewhere [7,25]. Patients were diagnosed by certified specialists in otorhinolaryngology and audiology. Tinnitus severity was assessed using the specific tinnitus questionnaire developed by Hallam and modified by Goebel and Hiller [69]. This questionnaire is suitable for repeated use with short intertest intervals [69]. Tinnitus duration was at least 6 months (mean duration 46.7 months; SD 41.1), the mean tinnitus-score was 46.8 (SD 14.9) (table 1). Patients with concomitant anticonvulsant drug treatment, unilateral hearing loss (defined as 15 dB minimum difference compared to the other ear) or middle ear pathologies were not included. All patients gave their written informed consent to take part in the study, which was approved by the local ethics committee.

### Functional and structural imaging for target detection

Functional neuroimaging data was assessed by [^18^F] deoxyglucose (FDG) positron emission tomography (PET) measurements (ECAT EXACT 47, Siemens). External acoustic stimulation was eliminated by plugging both ears hermetically.

**Figure 5:**
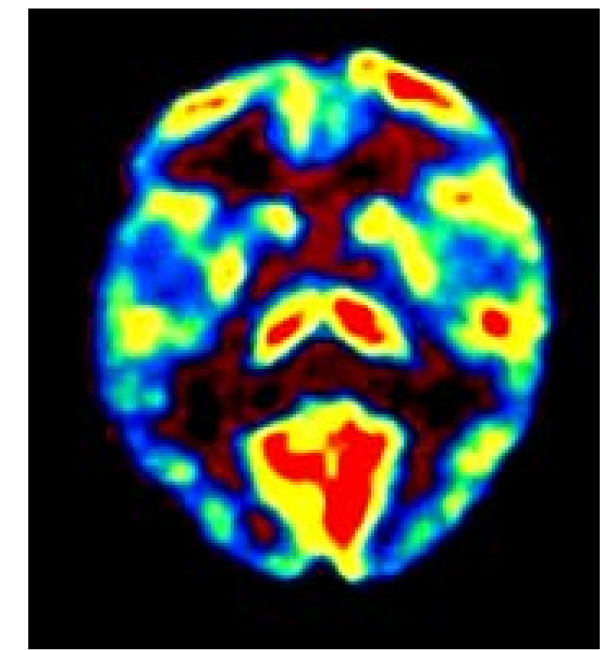
**FDG-PET of patient #1**. [^18^F] deoxyglucose (FDG) positron emission tomography (PET) had been performed in each patient before treatment. The area of hypermetabolic activity in the temporal cortex was chosen as target for TMS treatment. Here the FDG PET of patient #1 is displayed, where a transversal slice through the temporal brain region shows unilaterally increased metabolic activity in projection to the left auditory cortex.

**Figure 6:**
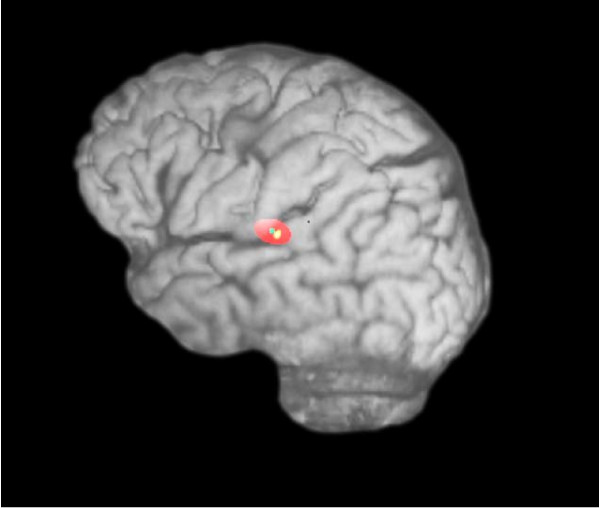
**Site of stimulation on a 3-D brain reconstruction**. The red area approximates the magnetic field on the brain surface, as computed by the neuronavigation system.

Only patients with a focal increase of FDG uptake in the region of the primary auditory cortex were included in this study (fig 5; table 1). Fusioning with structural MRI-data (MPRAGE, T1 weighted, 1.5 T Magnetom Symphony MR Scanner; Siemens) demonstrated that the area of increased activation was located within the superior temporal gyrus in all patients. This area was selected as a target for rTMS application (fig 6).

### rTMS treatment

A neuronavigational system used for neurosurgery was further developed and adapted for TMS (Vectorvision, BrainLAB AG, Heimstetten, Germany), to determine the coil localisation for stimulation, This technique offered the option to navigate the coil according to the patients' individual neuroimaging data and allowed real time visualisation of the magnetic field in relation to brain areas of interest. The focus of the magnetic field was directed at the area of the auditory cortex which showed maximal activation by FDG-PET, thus ruling out potentially confounding factors such as individual differences in skull-brain relations or variable location of cortical activation [24,70].

Patients were blinded to stimulation conditions and treated in a randomized cross-over design with 5 consecutive days of active treatment and 5 consecutive days of sham stimulation, separated by 9 days without TMS treatment. On each study day 2000 stimuli with a frequency of 1 Hz were administered using a MAGSTIM system and a figure-of-eight coil. For sham stimulation, a specific sham-coil system was used (MAGSTIM Co., Whitland, Dyfed, UK). Stimulation intensity was set at 110% motor threshold [25].

### Measurement of cortical excitability

Motor-evoked potentials (MEP) of the abductor digiti minimi (ADM) muscle of the right hand were recorded with surface electrodes, using a conventional EMG machine (Medelec MS 91A, England) with bandpass of 20 Hz tod 10 kHz. The signal was digitised at a frequency of 5 KHz and transferred into a laboratory computer for off-line analysis.

TMS was performed using a Bistim module, which was connected to two Magstim 200 stimulators (Magstim Co., Whiteland, Dyfed, UK). The figure-of-eight coil (outer diameter of each wing 90 mm) was held with the junction of the two wings tangential to the skull and the handle pointing backwards and ~45° away from the midline. Thus, the current induced in the brain was directed about perpendicularly to the assumed line of the central sulcus and therefore was optimal for activating the corticospinal pathways transsynaptically. The optimal coil position for stimulation was defined as the position above the left motor cortex for eliciting MEP of maximal amplitude in ADM with a slightly suprathreshold stimulus.

By reducing the stimulus intensity in steps of 1%, we defined the resting motor threshold (RMT) as the lowest intensity at which at least 4 of 8 consecutive MEPs were ≥ 50 μV in amplitude while the muscle being investigated was at rest. Audio-visual electromyographic feedback was provided to assess muscle relaxation. Active motor threshold (AMT) was determined as the lowest stimulation intensity that evoked a MEP ≥ 250 μV during voluntary abduction of the small finger in a minimum of 4 out of 8 consecutive trials. A constant level of voluntary contraction was maintained by audiovisual feedback of the EMG activity.

Cortical silent period (CSP) was measured in 10 trials at a stimulus intensity of 150% RMT with an inter-sweep interval of 5 s in the moderately active ADM (voluntary abduction with 30% of maximal force, monitored by audio-visual electromyographic feedback). CSP duration was defined as the interval between the end of the MEP and first reappearance of voluntary EMG activity. The measurements were made off-line on the non-rectified recording of every individual sweep and then averaged.

Intracortical excitability was measured using the paired-pulse paradigm consisting of a first subthreshold conditioning pulse followed by a second suprathreshold test pulse. The intensity of the first stimulus was set to 90% AMT, while the intensity of the suprathreshold test pulse was adjusted to produce an unconditioned MEP of ~1 mV. Inter-stimulus intervals (ISI) of 2, 3, 4, 5, 7, 8, 10, 15 and 20 ms were tested. Three blocks of trials were performed, each consisting of four randomly intermixed conditions presented 10 times each: the unconditioned test pulse and three conditions with the conditioning stimulus occurring at different intervals before the test pulse. The interval between sweeps was 4 s. The effect of conditioning stimuli on MEP amplitude at each ISI was determined as the ratio of the average amplitude of the conditioned MEP to the average amplitude of the unconditioned test MEP performed in the same block of trials.

Since it was known from previous studies that the conditioning stimulus has a suppressive effect on the control MEP at short ISIs (2–5 ms) and a facilitatory effect at longer ISIs (7–20 ms) [71], intracortical inhibition (ICI) and intracortical facilitation (ICF) were calculated across these intervals respectively [45,72].

Tinnitus complaints and motor cortex excitability were assessed at baseline (day 1), immediately after the last rTMS session (day 5) and three and six days post TMS treatment (days 8 and 11) for both stimulation conditions.

### Statistical analysis

Prior to conducting analyses on treatment effects, baseline values of excitability and tinnitus measures were compared by paired t-tests to check for potential carry-over effects. While the main interest is in analyzing the connection of change in intracortical excitability (ΔE) to change in tinnitus complaints (ΔTQ), first the absolute levels were analyzed using a separate analysis of variance models (ANOVA) including the explanatory factors "condition" (verum vs. sham) and "time" and their interaction together with the factor "treatment order". To test for a relationship between ΔTQ and ΔE, we performed a multiple correlation analysis, i.e. we regressed ΔTQ on the ΔE parameters for each measurement time separately and extracted the R^2^, which corresponds to the squared multiple correlation coefficient. This analysis was done separately for the active and the sham condition, to give multiple correlations for days 5, 8, and 11, separately for the verum and sham conditions. By contrasting these, the detection of contingency patterns caused by active treatment with the sham condition as a baseline was allowed for. For exploring patterns specific to single excitability measures, post hoc correlation analyses were performed. A MANCOVA testing for differences in ΔE between the verum and the sham condition, as well as consideration of all excitability measures simultaneously, provided significance screening to determine the measures to undergo post hoc analysis. We used a general linear model with multiple responses based on verum – sham differences in ΔE (baseline, day 11) with predictors "treatment order" (for adjustment) and the verum-sham difference in ΔTQ (baseline, day 11). Depending on the result, we conducted post-hoc separate correlation analyses of single excitability parameter (ΔE_CSP_, ΔE_ICI_, ΔE_ICF_) and ΔTQ for the verum condition to analyze in which direction the different excitability parameters changed.

## Authors' contributions

BL participated in the conception and the design of the study, carried out the excitability measurements and drafted the manuscript; TK participated in the design of the study, recruited the patients and performed audiologic measurements; JM participated in the design of the study and performed positron emission tomography; HB participated in the design of the study and performed the statistical analysis; PS participated in the design of the study and helped to draft the manuscript; GH participated in the study design and study coordination; PE conceived the study, participated in its design and coordination and helped to draft the manuscript. All authors read and approved the final manuscript.
